# Degradation of neural representations in higher visual cortex by sleep deprivation

**DOI:** 10.1038/srep45532

**Published:** 2017-03-31

**Authors:** Jia-Hou Poh, Michael W. L. Chee

**Affiliations:** 1 Centre for Cognitive Neuroscience Duke-NUS Medical School, 8 College Road, Singapore, 169857; 2 NUS Graduate School for Integrative Sciences & Engineering, 28 Medical Drive, Singapore, 117456.

## Abstract

A night of total sleep deprivation (TSD) impairs selective attention and is accompanied by attenuated activation within ventral visual cortex (VVC). However, finer details of how TSD compromises selectivity of visual processing remain unclear. Drawing from prior work in cognitive aging, we predicted that TSD would result in dedifferentiation of neural responses for faces and houses within the VVC. Instead, we found preservation of category selectivity. This was observed both in voxels highly selective for each category, and also across multiple voxels evaluated using MVPA. Based on prior findings of impaired attentional modulation following TSD, we also predicted reduced biasing of neural representations towards the attended category when participants viewed ambiguous face/house images. When participants were well rested, attention to houses (or faces) caused activation patterns to more closely resemble those elicited by isolated house (face) images than face (house) images. During TSD, attention to faces enhanced neural similarity to *both* target (face) and distractor (house) representations, signifying reduced suppression of irrelevant information. Degraded sensory processing reflected in reduced VVC activation following TSD, thus appears to be a result of impaired top-down modulation of sensory representations instead of degraded *selectivity* of maximally category sensitive voxels, or the dedifferentiation of neural activation patterns.

Anyone who has experienced a sleepless overnight journey will recall how difficult it can be to pick out a greeting friend from a crowd than if they were well rested. The neuroimaging counterparts of this ‘mental fog’ have been partially revealed in previous work on young adults that showed decrements in selective attention^1^,^2^,^3^ as well as distractor suppression^4^ after a night of total sleep deprivation. The reduced ventral visual cortex (VVC) activation accompanying these behavioural deficits has been attributed to reduced ability to exert top-down control on visual processing. A likely outcome of such loss of top down control would be degradation of the representation of visual information in visual cortex.

While degraded perceptual representation of stimulus information is intuitively appealing^5^ there is presently a lack of empirical evidence for it. There are also no clear models from sleep research on which to base a theoretically well-founded investigation into this possibility. In contrast, degraded sensory representations have been well studied in cognitive aging, to which sleep deprivation has been likened^6^,^7^. Aging related differences in the fidelity of neural representations have been suggested by computational modelling^8^,^9^ and are supported by neuroimaging findings in the ventral visual cortex^10^,^11^,^12^.

One mechanism that could explain the degradation of sensory representations is perceptual dedifferentiation where cortical responses to different stimulus categories become less distinct or more similar. This has been observed at the single voxel level^11^ as well as through multivariate pattern analysis (MVPA)^12^. Specifically, dedifferentiation of perceptual representations as a result of cognitive aging has been demonstrated in the form of reduced selectivity of individual VVC voxels normally exhibiting category-specific biases to faces and houses^11^. Loss of category selectivity has also been observed when inspecting spatial patterns of activation in groups of voxels using MVPA. Elderly participants’ activation patterns in response to different visual categories have been shown to be less separable, and such dedifferentiation was negatively correlated with fluid processing abilities^12^. In light of these findings, we predicted that we would find degraded sensory representation following TSD in the form of dedifferentiated VVC responses, either in individual category-selective voxels, or in altered neural activation patterns.

A second potential mechanism underlying degraded sensory representation is faulty top-down modulation of sensory representations in VVC. This explanation is related to the loss of distractor suppression in cognitive aging^13^,^14^ as well as following sleep deprivation^4^. In addition to enhancing activation to an attended category and suppressing activation to distractors, attention can bias activation patterns to be more similar to those of an attended target relative to its distractor^15^. Given the possibility of impairment in top-down modulation, we could expect attenuation of attention driven differentiation of neural patterns such that when participants view ambiguous images, the pattern of activation is not selectively biased towards that elicited by the relevant target category.

To examine if these mechanisms of degraded neural representation can explain perceptual deficits in sleep deprivation, we performed additional analyses on data from a previous study^4^, in which participants were instructed to attend to either faces or houses when viewing either isolated or ambiguous (i.e., overlapping) face-house images. All participants performed the task under conditions of sleep deprivation and rested wakefulness. We examined responses to individual face and house pictures at the single voxel and multi-voxels levels for the extent to which these would differentiate across the two visual categories, predicting that sleep deprivation would compromise this differentiation. We then examined responses to ambiguous face/house pictures when participants were told to attend to one or the other category, looking for a loss in biasing of signals across a patch of voxels, which would signify failure to inhibit the processing of the irrelevant category.

## Results

### Behavioural results

Participants performed a target detection task during the scan, requiring them to identify the presence of a target house or face image (presented at the start of each run). Task performance was evaluated using d’, and we performed a 2 × 2 × 2 ANOVA with factors of state (RW vs TSD), distractor presence (isolated vs ambiguous) and stimulus category (face vs house).

Participants’ performance was poorer during TSD than RW, evidenced by lower d’ (F(1, 19) = 21.37, *p* < 0.001, η_p_^2^ = 0.53). There was also a significant main effect of distraction, where performance was poorer for ambiguous face-house images than for isolated face or house images (F(1,19) = 72.83, *p* < 0.001, η_p_^2^ = 0.79). There was no significant main effect of stimulus category, (F(1,19) = 1.58, *p* = 0.224) indicating that detection accuracy was comparable for faces and houses.

**Figure 1:**
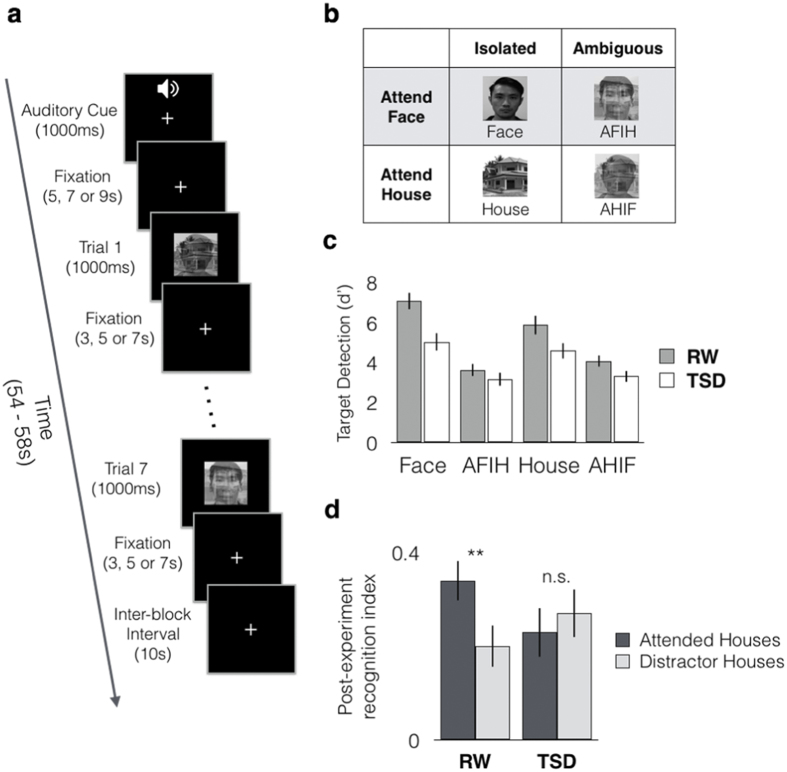
Experimental schematics and behavioural task performance. (**a**) Example of a single task block. Each run consisted of five blocks, one from each of the five conditions, presented in random order. An auditory cue at the start of each block, informed participants to attend to faces, houses, or to passively view the images. (**b**) Sample stimuli from each of the four task conditions (excluding passive view). (**c**) Target detection performance was poorer following TSD. (**d**) A recognition index was computed by subtracting mean rating of novel house stimuli from the mean rating of old house stimuli. Post-experiment recognition was such that under well-rested conditions, house images were better remembered when they were attended than if they were distractors to be ignored. This attentional modulation was not significant following sleep deprivation. Error bars indicate +/−1 SEM. **p < 0.01.

For the post-experiment recognition task, we observed a significant state by attention (AHIF vs AFIH) interaction (F(1,19) = 8.69, p = 0.008, η_p_^2^ = 0.31), where participants had higher recognition scores for houses during RW under the AHIF relative to the AFIH condition (*t*(19) = 3.59, *p* = 0.002, *d* = 0.80), but not during TSD (*t*(19) = −1.03, *p* = 0.315) (Fig. 1c). This supports the notion that following sleep deprivation there was a loss of selectivity such that task-irrelevant houses in the AFIH condition were attended and remembered.

### TSD did not affect category selectivity of category discriminating VVC voxels

**Figure 2:**
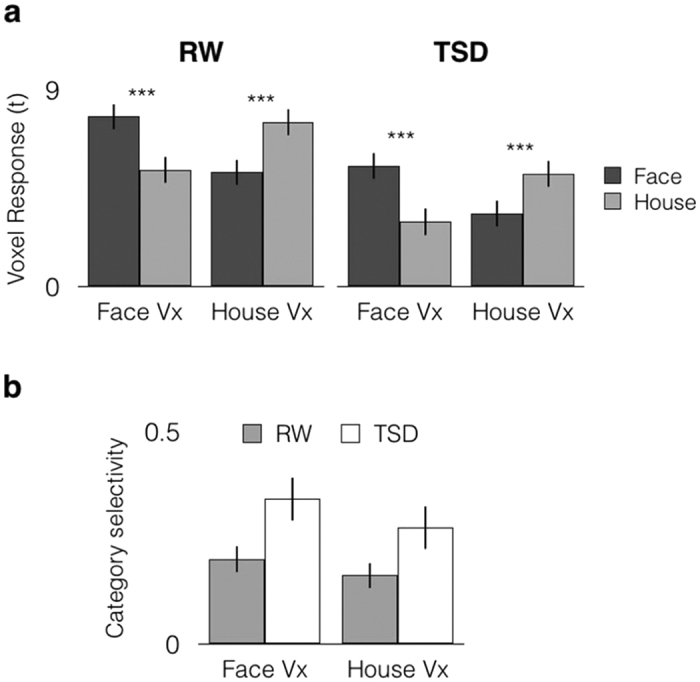
Category selective voxels responded more strongly to their preferred category and less strongly to their non-preferred category. (**a**) In both RW and TSD, face selective voxels (Face Vx) responded more strongly to isolated Face images (Face) than isolated House images (House), while house selective voxels (House Vx) responded more strongly to House than to Face. (**b**) Despite lower activation overall, normalized category-selectivity (t_preferred_ − t_non-preferred_)/(t_preferred  _ + t_non-preferred_) was preserved following sleep deprivation for both Face and House selective voxels. Error bars indicate +/−1 SEM. ***p < 0.001.

Category selectivity was studied through the examination of voxels showing the greatest response to each category. When examining response selectivity of the top 15 voxels, we found a significant voxel type (Face Vx, House Vx) by stimulus (Face image, House image) interaction (F(1,19) = 111.90, *p* < 0.001, η_p_^2^ = 0.86; Fig. 2a). The responses to a preferred stimulus were greater than for a non-preferred stimulus in both RW (Face Vx: *t*(19) = 6.18, *p* < 0.001, *d* = 1.39; House Vx: *t*(19) = 6.21, *p* < 0.001, *d* = 1.39) and TSD (Face Vx: *t*(19) = 5.74, *p* < 0.001, *d* = 1.29; House Vx: *t*(19) = 8.13, *p* < 0.001, *d* = 1.87).

To examine if there was dedifferentiation of category selective voxels following sleep deprivation, we compared category selectivity by computing the ratio of the difference in response amplitude for preferred and non-preferred stimulus categories relative to the sum of these measurements. A 2 × 2 ANOVA was conducted with state and voxel type as factors. There was a significant main effect of state (F(1,19) = 17.66, *p* < 0.001, η_p_^2^ = 0.48; Fig. 2b). Contrary to expectation, this was driven by greater selectivity in TSD than in RW (Face Vx: *t*(19) = −2.25, *p* = 0.036, *d* = 0.58; House Vx: *t*(19) = −2.31, *p* = 0.032, *d* = 0.52). This difference however, did not survive corrections for multiple comparisons (i.e. *p* < 0.025).

To ensure that our results were not an artifact of arbitrary voxel number thresholding, the same analysis was repeated over 10, 25 and 50 voxels. Results remained robust across the range of voxel numbers (Supplementary Figs S1 and S2).

### Distributed activation within the VVC distinguished Faces from Houses

**Figure 3:**
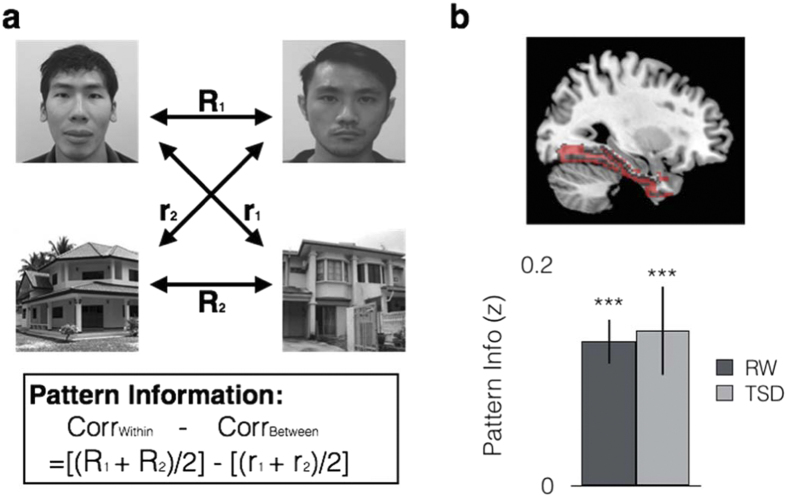
Cross-correlation analysis of pattern information. (**a**) A ROI was deemed to contain category information when the difference between its within- and between- category correlation was significantly greater than zero (16). (**b**) Results for cross correlation within the Ventral Visual Cortex (VVC). Pattern information in the VVC was significantly greater than zero in both RW and TSD. Error bars indicate +/−1 SEM. ***p < 0.001.

Cross-correlation^16^ was used to determine if distributed activation over multiple voxels within the VVC contained information that would distinguish between the two categories. Pattern information was significantly greater than zero in both RW (*t*(19) = 6.10, *p* < 0.001, *d* = 1.40) and TSD (*t*(19) = 3.89, *p* = 0.001, *d* = 0.89), indicating that VVC activation patterns contained information that distinguished isolated Faces from Houses even in the sleep deprived state that was associated with reduced activation amplitude (Fig. 3b). There was no significant difference in pattern information between RW and TSD (*t*(19) = −0.81, *p* = 0.858, *d* = −0.04).

To test whether this finding was driven by univariate differences in category-selective regions within the VVC (e.g., PPA or FFA), the same analysis was repeated after removing voxels showing univariate differences in category responses. Pattern information in both RW and TSD remained significantly greater than zero in both states (RW: *t*(19) = 4.43, *p* < 0.001, *d* = 1.02; TSD: *t*(19) = 2.76, *p* = 0.012, *d* = 0.64; Supplementary Fig. S3).

### Attention biased neural representation within the VVC in the well-rested state

Prior to probing whether attention biases neural representation towards attended categories comparably across states, we first checked for a systematic bias towards either category during passive viewing.

A direct comparison of similarity to Face and House images during passive viewing of ambiguous images showed no significant difference in either the RW or TSD conditions (RW: *t*(19) = 0.82, p = 0.422; TSD: *t*(19) = 1.72, *p* = 0.102). This suggests that there was no systematic bias towards either faces or houses during passive viewing of ambiguous images in either state.

**Figure 4:**
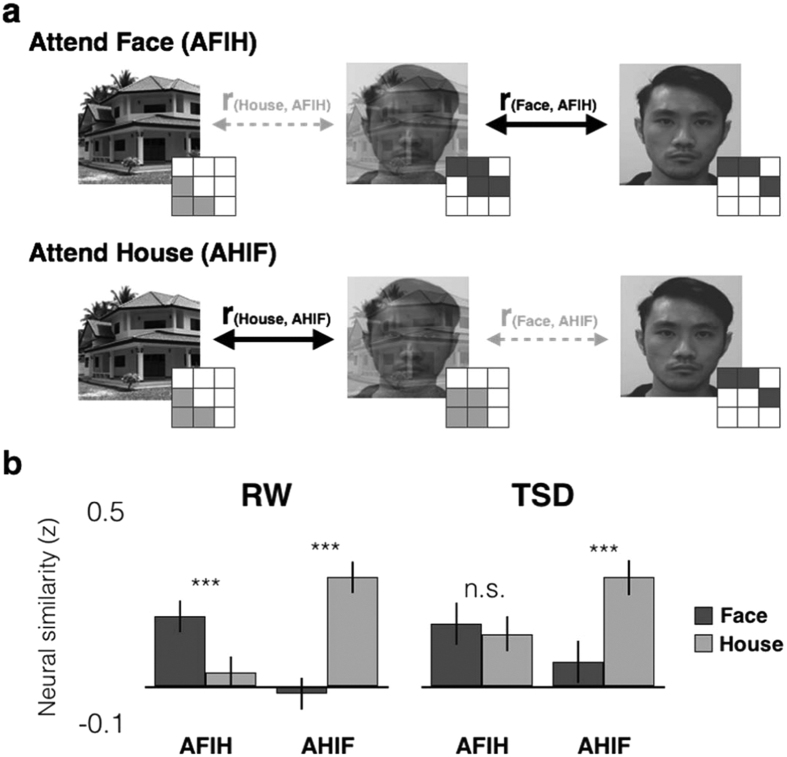
Selective attention to the target category in ambiguous images increases neural similarity to isolated targets. (**a**) Activation patterns elicited when viewing ambiguous face-house images were correlated with those associated with viewing isolated Face (Face template) or House (House template) images. With intact top down control of attention and distractor suppression, activation patterns during AFIH were expected to be more similar to those elicited by the Face template (i.e. r_(Face, AFIH)_ > r_(House, AFIH)_). Conversely in the AHIF condition, activations were expected to be more similar to those elicited by the House templates (i.e. r_(House, AHIF)_ > r_(Face, AHIF)_). (**b**) Attending to houses in ambiguous images elicited greater neural similarity to the House template than Face template in both RW and TSD. However, during TSD, attending to faces in ambiguous images did not elicit greater similarity to the Face template than the House template. Error bars indicate +/−1 SEM. ***p < 0.001.

We next determined if attention biased neural representation towards attended categories comparably across states. A 2 * 2 * 2 ANOVA was performed with state (RW & TSD), attention (AFIH & AHIF) and template category (Face & House) as factors (Fig. 4a). We observed a significant interaction between attention and category (F(1,19) = 75.73, *p* < 0.001, η_p_^2^ = 0.80). Subsequent followed-up paired comparisons showed that during RW, attending to faces in ambiguous images resulted in VVC patterns of activation more similar to those elicited by isolated Faces than to isolated Houses (*t*(19) = 4.07, *p* = 0.001, *d* = 0.91; Fig. 4b). Conversely, attending to houses while viewing ambiguous face-house images resulted in patterns that were more similar to those elicited by isolated Houses (*t*(19) = 4.26, *p* < 0.001, *d* = 0.95).

Similar paired-comparisons in TSD showed that while there was greater neural similarity to isolated Houses when attending to houses in ambiguous images (*t*(19) = 3.96, *p* = 0.001, *d* = 0.89; Fig. 4b), attending to faces in ambiguous images, did not result in greater similarity of patterns to those elicited by isolated Faces (*t*(19) = 0.35, *p* = 0.729).

### TSD impaired selectivity of attentional enhancement pointing to reduced ability to ignore competing, irrelevant information

To determine if impaired attentional modulation was responsible for the deficient representational bias towards the attended category, one-sample t-test was performed on the similarity measures. A value significantly greater than zero, would indicate representation enhancement relative to passive viewing.

Consistent with attention related enhancement of neural representations of the attended category, attending to faces (AFIH) enhanced similarity to the Face template relative to passive viewing. This was true both in RW (*t*(19) = 5.10, *p* < 0.001, *d* = 1.17) and in TSD (*t*(19) = 2.57, *p* = 0.019, *d* = 0.59).

Critically, a different pattern emerged for House representations in TSD. When participants attended to faces while viewing ambiguous images in RW, similarity to House templates did not significantly differ from passive viewing of these ambiguous images (*t*(19) = 0.93, *p* = 0.364) indicating adequate ability to suppress competing information. However, in TSD, attending to faces (AFIH) was accompanied by significant *enhancement* of house representations (*t*(19) = 2.88, *p* = 0.010, *d* = 0.66) suggesting greater processing of irrelevant house information following sleep deprivation.

## Discussion

We contrasted three measures of neural representation of faces and houses within the VVC during TSD and RW. Contrary to our prediction, sleep deprivation did not compromise category selectivity of ventral visual cortex activation at either the voxel or multi-voxels levels within the ventral visual cortex. However, extending results of prior work, we found that attention to faces in ambiguous images enhanced neural similarity to both target and distractor representations in TSD, signifying impaired ability to ignore competing information. This was supported by behavioural evidence for greater incidental encoding of task-irrelevant house distractors when participants were sleep deprived compared to when they were well-rested.

Most comparisons of the cognitive effects of sleep deprivation and aging have highlighted reduced prefrontal function^6^,^7^. However, attenuated activation of the ventral visual cortex of sleep-deprived persons has also been repeatedly observed^1^,^2^,^3^,^17^ and resembles similar observations in older adults^18^,^19^. These latter observations support a theoretical framework wherein impaired sensory processing contributes to deficits found in cognitive aging^19^,^20^,^21^,^22^ by reducing the signal-to-noise ratio of perceptual representations^9^,^23^. The present work was inspired by these observations, as well as subsequent findings of degraded neural representation of visual stimuli both at the voxel^11^ and pattern level in older adults^10^,^24^. However, in the present study, only impaired attentional modulation was found to be occurring in TSD. This is consistent with a prior finding demonstrating a difference between TSD and cognitive aging despite surface similarities^25^.

Contrary to expectation, category selectivity of voxels responding most strongly to faces or houses was preserved in TSD. Further, category information widely distributed across the VVC^16^ was also preserved in TSD despite the reduction in average VVC activation amplitude. Critically, category information was assessed as intact even after excluding the peak voxels that showed category selectivity, indicating that preserved selectivity extended beyond a small sample of peak voxels.

While it seems counter-intuitive that reduced signal amplitude within the VVC did not lead to reduced pattern information, several studies have shown that lower fMRI signal amplitude is compatible with preserved^26^ or even enhanced information representation^27^. The latter may occur when reduced activation arises from ‘sharpening’ of neural responses such that sparser, but more feature relevant representations are recruited^28^. Lower activation amplitude during sleep deprivation may correspond to the reduced ability to keep neural circuits in the ‘up’ state^29^. This could result in a reduction in functional redundancy of activation that could otherwise hold up function under TSD^30^. The preserved activation of sufficient circuitry but being bereft of additional capacity to support cognitive control of perceptual representations could explain why there was intact category selectivity for isolated images but inadequate neural representation for processing ambiguous images.

In using ambiguous face-house pictures, we were able to examine how attention biases neural representations while keeping perceptual input constant^15^,^31^,^32^. In the well-rested state, attention biased activation patterns to more closely resemble those elicited by the attended category compared to the unattended category. Adequate distractor suppression in the rested state was evidenced by parity in representation similarity between the ignored and passively viewed category.

Following sleep deprivation, this biasing effect of selective attention was not observed when participants were required to attend to faces and ignore houses. Compared to the passive viewing condition, we showed that attending to faces in TSD, not only resulted in enhanced face representation, but also enhanced house representation, suggesting a deficiency in the suppression of distractor information. As with our findings concerning category selectivity, the pattern-based measure of attenuated distractor suppression extends previous work at the univariate level^4^ in showing that the neural substrate for this deficit extends to voxels other than those within classic category selective VVC, e.g. the PPA.

The suppression of competing irrelevant information can be dissociated from attentional enhancement, and loss of the former has been associated with lower working memory performance^14^,^33^ and reduced ability to multi-task^34^ in older adults. Consistent with these findings, we previously reported that when sleep deprived, participants’ memory for ignored Houses in ambiguous images was comparable to memory for attended Houses^4^.

In the present study, the enhanced representation for distractor house information could arguably be represented as ‘distractor excitation’ suggesting a non-selective enhancement of distractor information. However, in our comparison with a passive viewing baseline, measures of similarity could also include correlations driven by attention^35^, resulting in higher correlation values in tasks requiring active monitoring for a target. Non-selective enhancement is functionally equivalent to impaired distractor suppression in that irrelevant information receives more processing than intended. Our behavioural findings support this interpretation, in that following TSD, participants showed similar levels of recognition for both attended and unattended houses.

A potential limitation of our account, lies in the asymmetric deficiency in distractor suppression, which was only observed when participants attended to faces (and failed to ignore houses), but not when participants attended to houses (and successfully ignored faces).

We note that several prior studies examining attentional modulation using Face and House images have also focused their analysis on the scene-selective PPA^36^,^37^. A study examining both PPA and FFA, observed a similar asymmetry in functional activation as that observed here^32^. The reasons for this are presently unclear. A possible explanation could lie in a difference in processing demands of faces and houses. Processing of faces tends to occur in a holistic manner^38^,^39^, and this could thus facilitate the requirement to enhance or suppress face representations. In contrast, attentional modulation of house images might be more demanding due to the need for local processing, particularly when features of such images are spatially discontinuous, or occupy greater spatial extent.

A topic that would benefit from future research would be the extent to which visual search in natural scenes is perturbed in the sleep deprived state. Most real world visual search and mnemonic tasks like finding a friend in a crowd require distractor suppression. Beyond simply being more ecologically valid, prior work has shown that when processing capacity is diminished as it is in cognitive aging, one becomes less able to process visual information in context^40^. As such, a possible remedy could lie in providing a supportive rather than misleading context that could significantly reduce memory interference^41^.

Using a combination of univariate and correlation-based MVPA approaches, we clarified that the altered sensory processing reflected by reduced VVC activation in sleep-deprived persons appears to be a result of impaired top-down modulation of sensory representations of irrelevant information, rather than degraded category selectivity or neural dedifferentiation for isolated objects.

## Materials and Methods

### Participants

Twenty-two healthy right-handed participants were studied after giving informed consent. All participants were selected based on a web-based questionnaire, and were screened based on the following requirements: (1) right-handed, (2) regular sleeping habits, (3) sleep duration of >6.5 h per night, (4) not on long term medication, (5) no symptoms or history of sleep disorders, (6) no history of psychiatric or neurologic disorders, and (7) drank less than 3 caffeinated drinks per day. The experimental protocol was approved by the Institutional Review Board of the National University of Singapore. All methods were performed in accordance with the relevant guidelines and regulations. Data corruption resulted in a loss of two participants’ records. Therefore, this report is based on data from 20 participants (11 females, M = 20.7 years, SD = 1.5 years).

### Study procedure

Participants visited the laboratory on three separate occasions, each separated by approximately 1 week. In the first session, participants were briefed on the study protocol and were informed about the study’s requirements. At the end of this session, participants were provided with a wrist actigraph (Actiwatch, Philips Respironics, USA), which they were required to wear throughout the duration of the study.

Participants were scanned twice, once during rested wakefulness (RW), and once after 24 h of total sleep deprivation (TSD). RW scans took place at 0800 h, and TSD scans took place at 0600 h. Test timings were selected to capture the circadian nadir^42^,^43^. The effects of sleep deprivation describe here thus represent a combination of circadian and homeostatic effects^44^,^45^. The order of scans was counterbalanced across subjects, and sessions were scheduled approximately 1 week apart. They were required to abstain from caffeine, medication and alcohol 24 hours prior to their experimental session.

For the TSD session, participants arrived at the laboratory in the evening and were monitored in the laboratory till the scanning session the next morning. Participants were seated in a well-lit room under the observation of a research assistant, and were allowed to engage in non-strenuous activities, such as reading and watching videos. Every hour throughout the TSD session, they performed the Psychomotor Vigilance Task^46^, and indicated subjective sleepiness on the Karolinska Sleepiness Scale^47^.

All participants were required to maintain a regular sleep-wake pattern (6.5–9 hours a night, sleeping before 0030 h and waking before 0900 h) for the duration of the study. Sleeping patterns were measured using actigraphy and sleep diaries.

### Experimental task and temporal organization

Task conditions were organised into blocks, where participants viewed a stream of images that comprised isolated house images (House), isolated face images (Face), or ambiguous (overlapping) face-house images in which either the house or the face had to be attended (Attend Face Ignore House (AFIH) or Attend House Ignore Face (AHIF; Fig. 1a). A target face and a target house image were presented to participants prior to each experimental run, and an auditory cue at the start of each block signalled whether participants were required to detect the target face or target house. They were required to respond with a button box held in their right hand when a target image appears (except during passive viewing blocks).

A total of 10 functional runs were conducted during each experimental session. Each run contained 5 randomised blocks, each comprising trials from one of five conditions (Face, House, AHIF, AFIH and Passive view). At the start of each block, a 1 s auditory cue was delivered instructing participants to attend to faces (Face – Isolated Face images and AFIH – ambiguous face-house images), to attend to houses (House – Isolated House images and AHIF – ambiguous face-house images) or to passively view ambiguous face-house images (Fig. 1b). A total of 7 images were presented within each block, and each image was presented for 1 s. The ISI was 3, 5 or 7 s. Each block contained one or two targets. Each experimental run lasted 252 s, and total duration of each scan session was approximately 42 mins.

### Post experiment memory evaluation

At the end of each session, a recognition task was administered outside the scanner. Participants were shown 200 house images (100 novel, 100 old), and were required to indicate on a 4-point scale whether the image shown was 1: Definitely New; 2: Probably New; 3: Probably Old; or 4: Definitely Old. Old images shown included 8 targets, and 23 non-targets from each condition (i.e. House, AFIH, AHIF & Passive view). A recognition index was calculated by subtracting mean rating of novel house stimuli, from the mean rating of old house stimuli for each participant^13^,^48^. Ratings were separately derived for AFIH and AHIF conditions. If selective attention and distractor suppression are adequately engaged when viewing ambiguous pictures, we expected recognition of house stimuli to be greater when houses were attended relative to when they were not.

To replicate behavioural findings from the original work with the current subset of participants, similar behavioural analysis was conducted. Analysis for both the experimental and post-experiment memory task was performed using repeated measures ANOVA, and Bonferroni correction was applied based on the number of paired comparisons performed within each model.

### Imaging parameters

Images were acquired on a 3-Tesla Tim Trio system (Siemens, Erlangen, Germany). Functional images were obtained using a gradient EPI sequence with 36 axial slices (slice thickness 3 mm with 0.3 mm inter-slice gap), using the following parameters: TR 2000 ms; TE 30 ms; flip angle 75 degrees; FOV 192 × 192 mm; voxel size 3 mm isotropic). A high-resolution anatomical reference image was acquired using an MPRAGE sequence (TR 2300 ms; TI 900 ms; flip angle 9 degree; voxel size 1 mm isotropic).

### Image analysis

In the preprocessing stage, the first four volumes of each run were discarded for T1 stabilization, and all images were realigned to the first image of the functional run using rigid body transformation. Slice-time correction was performed using trilinear and sinc interpolation implemented in SPM2 (Wellcome Department of Cognitive Neurology, London, UK). Individual T1 scans were reconstructed into a surface representation, and functional data were co-registered using the reconstructed cortical surface^49^ using FreeSurfer (http://surfer.nmr.mgh.harvard.edu). The co-registered images were then transformed into MNI152 space, and were smoothed with a 4 mm FWHM smoothing kernel. Details of this processing pipeline can be found in Yeo, *et al*.^50^.

Functional images for both RW and TSD scans were then analysed in SPM8 (Wellcome Department of Cognitive Neurology, London, UK) using a GLM with the following predictors: Face cue, House cue, Passive cue, Face, House, AFIH, AHIF and Passive view. Incorrect trials, missed trials, and target trials were modelled as regressors of no interest. Regressors were created by convolving relevant events with a canonical hemodynamic response function (HRF). Motion parameters were also included as covariates in the GLM.

All fMRI analyses were performed within predefined VVC ROI, and Bonferroni correction was applied based on the number of paired comparisons performed within each model.

### ROI definition

The ventral visual cortex (VVC) region-of-interest (ROI) was defined anatomically using the AAL atlas^51^. The ROI encompassed the (bilateral) regions labelled ‘Fusiform’, ‘Parahippocampal’ and ‘Inf Temporal’.

To address the possibility that pattern information could be driven by univariate differences between experimental conditions, we ran an additional analysis where voxels showing univariate differences were excluded. This was created through exclusive masking of significant voxels in the Face > House and House > Face contrast at an uncorrected threshold of *p* < 0.001. This procedure was repeated for each participant.

### Peak voxel analysis

To examine dedifferentiation of visual sensory responses following TSD, we identified voxels (extent: 10, 15, 25 and 50 voxels) showing the greatest response to isolated Face images (Face voxels) and isolated House images (House voxels) across both RW and TSD, based on Face > Baseline and House > Baseline contrasts.

We obtained the average *t*-value of these voxels and contrasted the response magnitude to a preferred stimulus (e.g., response of Face voxels to a face image) with the response to a non-preferred stimulus (e.g., response of Face voxels to a house image). Category selectivity was computed as the difference between a given voxel’s response to preferred and non-preferred stimuli, relative to the summed activation of both stimulus types, i.e., (t_preferred_ − t_non-preferred_)/(t_preferred_ + t_non-preferred_).

### Multivariate Pattern analysis – Correlation-based neural similarity

Information representation within the VVC was measured using correlation-based similarity analysis^52^,^53^. To establish that face-house category information was present in the VVC, we compared activation patterns for *isolated* face and house images using cross-correlation^16^. Herein, data were split into odd and even runs, yielding two pairs of within-category correlations (Face_odd_ & Face_even_; House_odd_ & House_even_) and two pairs of between category correlations (Face_odd_ & House_even_; House_odd_ & Face_even_) Category discrimination was inferred from the difference between within-category and between-category correlations (Fig. 3a).

To examine attentional modulation, mean activation patterns were first derived separately for isolated Face and House images. These were then used as ‘templates’ for subsequent comparisons with activation patterns elicited by ambiguous face-house images (Fig. 3). Pair-wise correlations were computed between the template patterns and activation patterns elicited during 3 conditions: Passive viewing, AFIH & AHIF.

Subsequent analyses were premised on the notion that attention would enhance activation patterns representing the attended category, such when attending to Houses in ambiguous images, activation patterns would be biased to more closely resemble the House templates rather than Face templates, the converse being expected for attention to Faces^15^. For computation, the similarity to templates derived from passive viewing of ambiguous images were subtracted from correlation values obtained when evaluating AFIH and AHIF conditions (i.e. r[Face, AFIH] − r[Face, Passive], r[House,AFIH] − r[House, Passive], … etc).

## Additional Information

**How to cite this article**: Poh, J.-H. and Chee, M. W.L. Degradation of neural representations in higher visual cortex by sleep deprivation. *Sci. Rep.* **7**, 45532; doi: 10.1038/srep45532 (2017).

**Publisher's note:** Springer Nature remains neutral with regard to jurisdictional claims in published maps and institutional affiliations.

## Supplementary Material

Supplementary Materials
