# Supplementary material for: Degradation of neural representations in higher visual cortex by sleep deprivation
Source: Sci Rep. 2017 Mar 31;7:45532. doi: 10.1038/srep45532 (PMC5374525; doi:10.1038/srep45532)
Supplement: Supplementary Materials [file srep45532-s1.pdf]

Supplementary Materials

Degradation of neural representations in higher visual cortex by sleep deprivation

Jia-Hou Poh[1, 2] and Michael W.L. Chee[1] *

[1]Centre for Cognitive Neuroscience

Duke-NUS Medical School

8 College Road, Singapore 169857

[2]NUS Graduate School for Integrative Sciences & Engineering

28 Medical Drive, Singapore 117456

*Corresponding Author: Michael W.L. Chee (michael.chee@duke-nus.edu.sg)

Mailing Address: Centre for Cognitive Neuroscience, Duke-NUS Graduate Medical School, 8 College Road, Singapore 169857. Fax: +6562218625

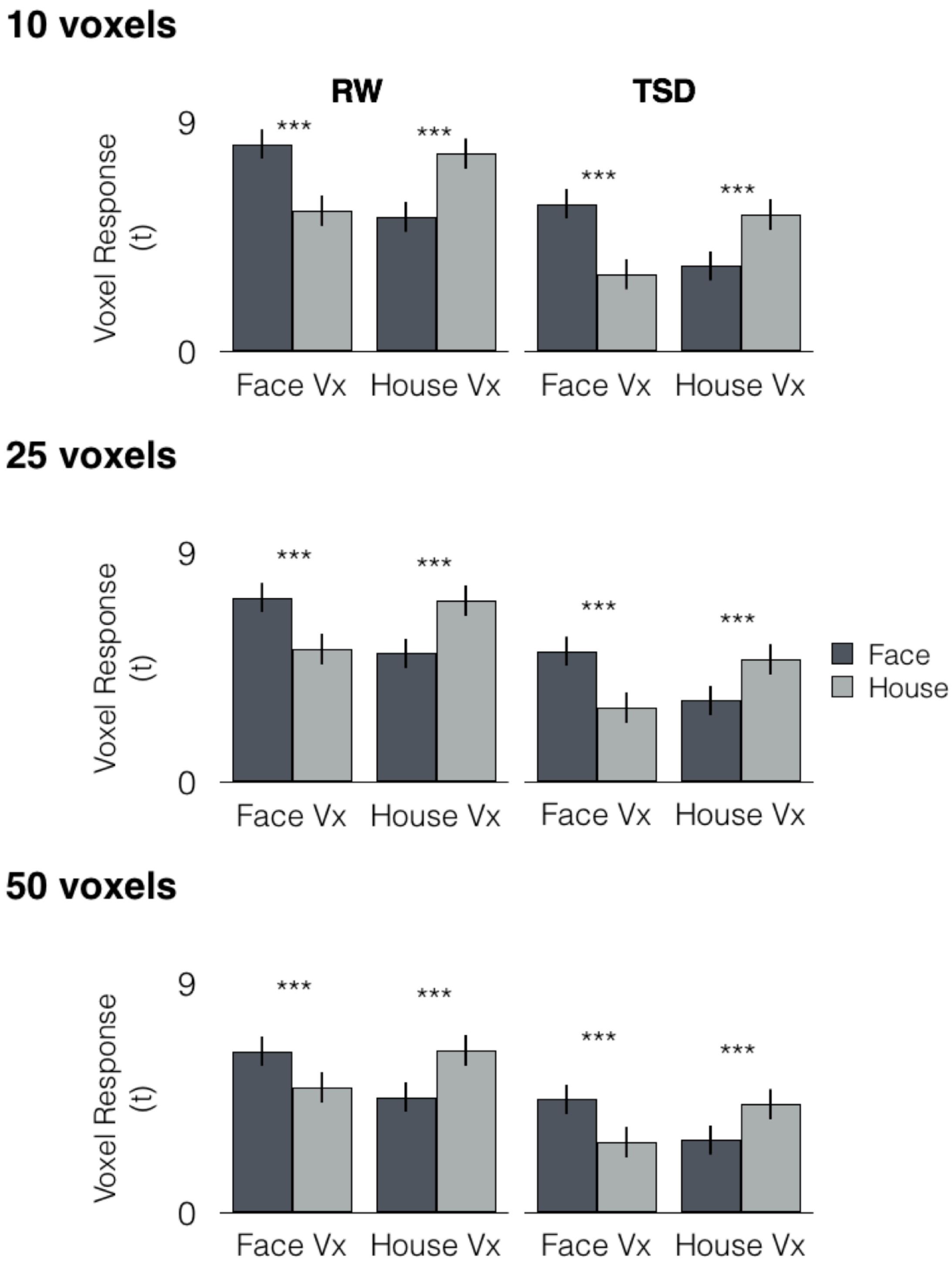

Fig S1. Category selective voxels responded more strongly to their preferred category and less strongly to their non-preferred category across a range of voxel numbers.

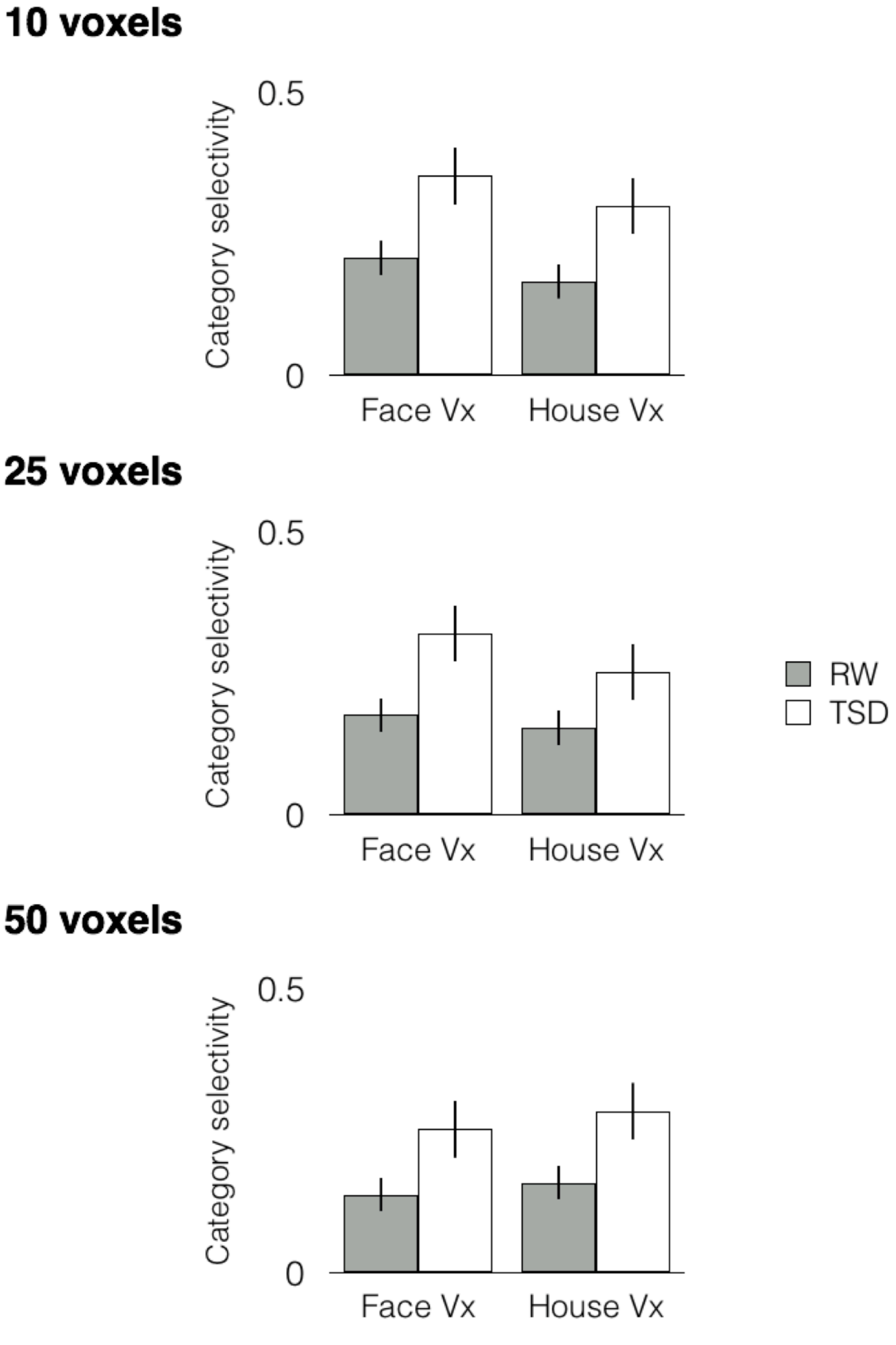

Fig S2. Normalized category selectivity was preserved following TSD across a range of voxel numbers.

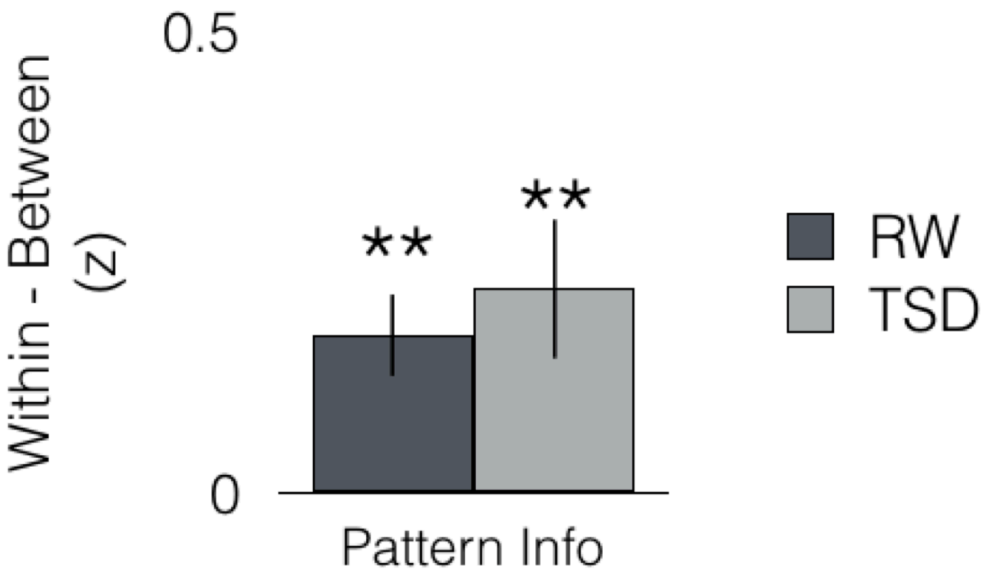

Fig S3. Cross-correlation analysis of pattern information, excluding voxels showing univariate differences at $p < .001$. Even after excluding voxels showing univariate differences, pattern information in the VVC was significantly greater than zero in both RW ($t(19) = 3.96$, $p = .001$) and TSD ($t(19) = 4.09$, $p = .008$). Error bars indicate +/- 1 SEM. ** $p < .01$.
